# Transmembrane Self-Assembled Cyclic Peptide Nanotubes Based on α‐Residues and Cyclic δ‐Amino Acids: A Computational Study

**DOI:** 10.3389/fchem.2021.704160

**Published:** 2021-07-27

**Authors:** Alexandre Blanco-González, Martín Calvelo, Pablo F. Garrido, Manuel Amorín, Juan R. Granja, Ángel Piñeiro, Rebeca Garcia-Fandino

**Affiliations:** ^1^ Departamento de Química Orgánica, Center for Research in Biological Chemistry and Molecular Materials, Universidade de Santiago de Compostela, Campus Vida s/n, Santiago de Compostela, Spain; ^2^ Departamento de Física Aplicada, Facultade de Física, Universidade de Santiago de Compostela, Santiago de Compostela, Spain

**Keywords:** self-assembling cyclic peptide nanotubes, transmembrane channels, molecular dynamics simulation, ion transport across lipid membrane, confined water

## Abstract

Self-assembling cyclic peptide nanotubes have been shown to function as synthetic, integral transmembrane channels. The combination of natural and nonnatural aminoacids in the sequence of cyclic peptides enables the control not only of their outer surface but also of the inner cavity behavior and properties, affecting, for instance, their permeability to different molecules including water and ions. Here, a thorough computational study on a new class of self-assembling peptide motifs, in which δ-aminocycloalkanecarboxylic acids are alternated with natural α-amino acids, is presented. The presence of synthetic δ-residues creates hydrophobic regions in these α,δ-SCPNs, which makes them especially attractive for their potential implementation in the design of new drug or diagnostic agent carrier systems. Using molecular dynamics simulations, the behavior of water molecules, different ions (Li^+^, Na^+^, K^+^, Cs^+^, and Ca^2+^), and their correspondent counter Cl^−^ anions is extensively investigated in the nanoconfined environment. The structure and dynamics are mutually combined in a diving immersion inside these transmembrane channels to discover a fascinating submarine nanoworld where star-shaped water channels guide the passage of cations and anions therethrough.

## Introduction

The translocation of different species between the compartmentalized inner regions of living cells and their environment plays a core role on their viability and probability of survival. Uncontrolled exchange of matter is equivalent to a death sentence for the cell. The exquisite control of nature on this dynamic equilibrium in biological ion channels and pores provides a source of inspiration for scientists who use natural structures and functions as a reference to be mimicked and eventually improved (Tagliazucchi and Szleifer, 2015; Bogdanowicz et al., 2016).

**FIGURE 1:**
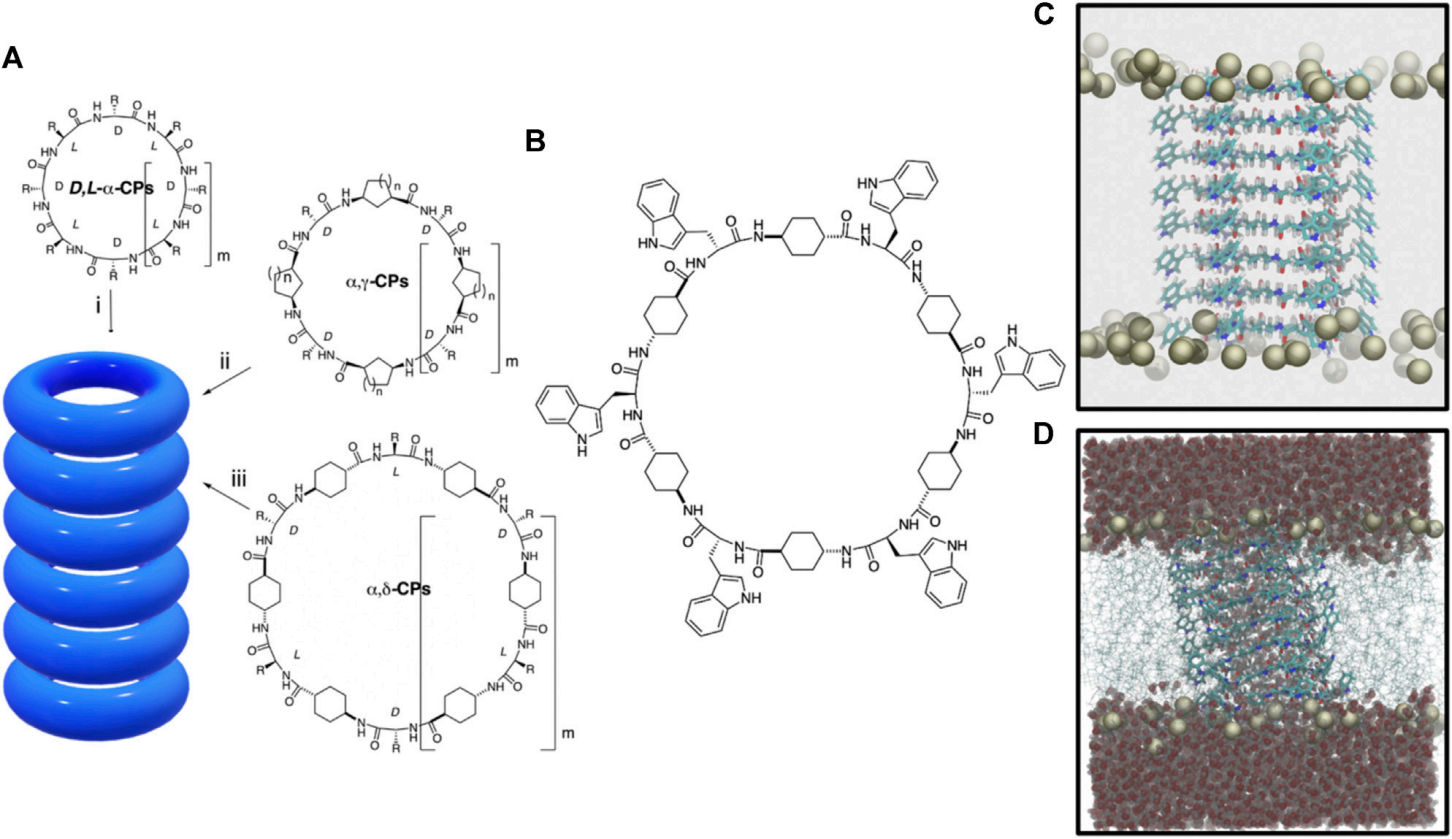
**(A)** Peptide nanotube models formed by the stacking of cyclic peptides of different types: (i) *D*,*L*-α-CP, (ii) CPs containing γ-Acas (α,γ-CPs), and (iii) CPs containing δ-Acas (α,δ-CPs). **(B)** *c*-[(*L*-Trp-δ-Ach-*D*-Trp-δ-Ach-)_3_] CP used in this work. **(C)** Snapshot of the initial α,δ-SCPN structure for the MD simulation. Only the phosphorous atoms of the lipids are shown. **(D)** Final structure (t = 200 ns) for the blank simulation (without ions), where also the lipid chains and the water molecules are shown.

Numerous supramolecular structures have been designed to replicate the specific functions of natural transport systems, in terms of affinity, efficiency, stability, and selectivity (Zheng et al., 2021). Among other examples, self-assembling cyclic peptide nanotubes (SCPNs) have emerged as attractive transmembrane channel mimetics due to their cylinder shape and expected biocompatibility (García-Fandiño et al., 2012b; Rodriguez-Vazquez et al., 2014; Garcia-Fandiño et al., 2017; Claro et al., 2018; Rodríguez-Vázquez et al., 2017). These systems were devised in 1974, but it was not until 1993 that they were first synthesized (De Santis et al., 1974; Ghadiri et al., 1993). The original SCPNs were comprised of *D*- and *L*-α-amino acids that adopted a flat conformation in which the amide groups (NH and CO) lie perpendicular to the plane of the ring (Figure 1A). (De Santis et al., 1974; Bong et al., 2001) This special arrangement allows the formation of hydrogen bonds (H-bonds) between contiguous cyclic peptides (CPs) owing to the complementarity between H-bond donor and acceptor groups on both sides of each disc structure. Thus, upon CP stacking, the interior of the assembly remains empty, with all the side chains exposed on the external surface of the cylindrical structure. One of the main advantages of these rod-shaped peptide materials is the simplicity with which their inner and outer properties can be easily modulated upon appropriate selection of the CP sequence; while the number and type of amino acids determine the internal diameter of the nanotube, the characteristics of its outer surface depend on the properties of the side chains of the amino acids. In this sense, the design of SCPNs that interact with the hydrophobic part of biomembranes has been carried out through the appropriate selection of amino acids in the basic peptide subunit (Ghadiri et al., 1994).

Most SCPNs have a hydrophilic inner cavity, designed to allow the transport of hydrophilic species (Brea et al., 2010). However, since the synthesis of the first *D*,*L*-α-SCPNs, SCPNs using CPs composed by different types of nonnatural amino acids have been developed, obtaining structures with more specific behavior (Montenegro et al., 2013). For example, β-SCPNs (Seebach et al., 1997; Clark et al., 1998a) are characterized by leaving all the NH groups in one side of the CPs and all the CO groups on the other. The resulting macrodipole leads to exotic properties such as a significant piezoelectricity (Tabata et al., 2019). Cyclic γ-amino acids (*cis*-γ-aminocycloalkanecarboxylic acids, γ-Acas) of appropriate chirality, both alone (Chen et al., 2020) or alternated with α-amino acids (α,γ-CPs), can also form nanotubes (α,γ-SCPNs) (Amorín et al., 2003; Garcia-Fandino et al., 2012), where the β-carbon of the cycloalkane moiety is oriented toward the lumen of the cylinder, influencing the internal properties of the nanotube and opening the possibility of inner functionalization (Figure 1A). The first steps toward the preparation and characterization of α,γ-SCPNs with chemically functionalized lumens have already anticipated promising results in this area (Reiriz et al., 2009; Calvelo et al., 2015; Rodríguez-Vázquez et al., 2017; Calvelo et al., 2019).

The behavior of water molecules and ions locked within the narrow confines of a subnanometric hydrophobic pore is very different from that of the macroscopic world. Both *in vitro* and *in silico* studies indicate that nanoconfined water inside SCPNs adopts a highly ordered structure and it shows a much slower free diffusion compared to its bulk form (Granja and Reza Ghadiri, 1994; Clark et al., 1998b; Sánchez-Quesada et al., 2001; García-Fandiño et al., 2009; García-Fandiño et al., 2012a; Montenegro et al., 2013). This seems to be modulated by several factors such as the channel inner radius and its hydrophobicity (Montenegro et al., 2013; Rodríguez-Vázquez et al., 2016). An increase in the hydrophobic character of the nanotube can be achieved using the *trans*-4-aminocyclohexanecarboxylic acid (δ-Ach) as a building block. The resulting nanotubes, α,δ-SCPNs (Lamas et al., 2018), exhibit a more hydrophobic internal cavity due to the presence of two methylene groups of each cyclohexyl moiety oriented toward the inner of the cylinder (Figure 1A). The presence of hydrophobic regions of transmembrane channels has been found to play an important role in controlling water and ion diffusion (Aryal et al., 2015; Rao et al., 2018; Yazdani et al., 2020), making α,γ- and α,δ-SCPNs especially attractive for their potential implementation in the design of new drug or diagnostic agent carrier systems. While several studies have been carried out to characterize transmembrane α,γ-SCPNs (García-Fandiño et al., 2012a; Calvelo et al., 2015; Garcia-Fandiño et al., 2016; Calvelo et al., 2019), biomimetic pores based on α,δ-SCPNs remain mostly uncharacterized.

Since experimental measurement about confined molecule properties in the nanoscale is difficult (Rodríguez-Vázquez et al., 2017), molecular dynamics (MD) simulations play a key role in the description of these systems (Lynch et al., 2020). Recent studies carried out in our group showed that α,δ-SCPNs are stable and theoretically able to translocate water and cations when inserted into a lipid bilayer (Calvelo et al., 2021). However, the behavior of the water molecules and the ions inside these structures has not been deeply examined.

This study aims to explore in detail the internal properties of transmembrane α,δ-SCPN channels, evaluating and carefully investigating the structural and dynamical behavior of water, LiCl, KCl, NaCl, CsCl, and CaCl_2_ inside this type of nanotubes. The confinement effect on the structure and dynamics of water and ions in the lumen of these pores is thoroughly analyzed. The simulation results will provide new insights, useful for a better design of cyclopeptide-based structures, allowing more rational constructions for improving the transmembrane channel activity.

## Materials and Methods

The α,δ-SCPN studied in this work is composed of eight CPs, long enough to traverse a previously equilibrated POPC membrane model (Calvelo et al., 2020). Each ring was made up of six α-amino acids (3 *L*- and 3 *D*-residues) and six δ-Acas acid residues {*c*-[(*L*-Trp-δ-Ach-*D*-Trp-δ-Ach-)_3_], Figure 1B}. The starting geometries of the employed CPs were taken from previous works (Calvelo et al., 2021), which suggested a preferred parallel disposition of the β-sheet (Figure 1C). After nanotube insertion into the POPC bilayer, the complete system was solvated. Water molecules in the hydrophobic region of the tails and also inside the SCPN were removed, so that, in the first step of the simulation, the channel was completely dry (Figure 1D). The resulting systems were ionized using different salt solutions (LiCl, NaCl, KCl, CsCl, and CaCl_2_ at 0.15 M, respectively). One extra blank simulation, for control, was carried out without ions. Thus, a total of six systems were prepared.

TIP3P (Price and Brooks, 2004) was chosen as the water model, and the parameters developed by Joung et al. (Joung and Cheatham, 2008) were selected to describe the ions Li^+^, Na^+^, K^+^, Cs^+^, Ca^2+^, and Cl^−^, as they are known to solve some crystallization issues observed with classical parameters at relatively high concentrations. For the standard residues, the AMBER99SB-ILDN force field parameters were chosen (Lindorff-Larsen et al., 2010). For the nonstandard amino acids, RESP/6-31G(d) charges were derived, similar to those used in the development of AMBER force fields, and van der Waals parameters were obtained from the GAFF force field, all taken from a previous work (Calvelo et al., 2021). The parameters for the lipid of choice, POPC, were taken from Lipidbook (Klauda et al., 2010; Jämbeck and Lyubartsev, 2012a; Jämbeck and Lyubartsev, 2012b).

All simulations were performed with the GROMACS 2019.3 package (Abraham et al., 2015). All systems were first minimized, followed by an unrestrained production run of 200 ns, with a time step of 2 fs. No specific restraints were applied to the peptides at any step. An NPT ensemble was employed at 1 bar using a semi-isotropic Parrinello–Rahman barostat (Parrinello and Rahman, 1981) and at 300 K maintained with a V-rescale thermostat (Bussi et al., 2007). The LINCS algorithm was employed to remove all bond vibrations (Hess et al., 1997). Electrostatic interactions were calculated using the PME method with a cutoff of 1.0 nm and a grid spacing of 0.12 nm (Essmann et al., 1995). Van der Waals interactions were calculated using a 1.0 nm radius cutoff.

The data obtained from the MD simulations were treated and analyzed with GROMACS and specific codes developed in Python. MDAnalysis (Michaud-Agrawal et al., 2011; Gowers et al., 2016) was employed to preprocess the trajectories, to calculate the water and ion survival probability [*P*(τ)] in the SCPN, and the radial distribution functions [*g*(r)]. NumPy (Harris et al., 2020) and Pandas (Mckinney, 2010) were also used to treat and organize data, and Matplotlib (Hunter, 2007) was used for graphical representations. The snapshots and animations from trajectories were made using VMD (Humphrey et al., 1996). *g*(r) was employed as implemented in MDAnalysis, while *P*(τ) was obtained from the following equation:

$$P\left(\tau \right)=\;\frac{1}{T}\sum_{t=1}^{T}\frac{N\left(t,\;t+\tau \right)}{N\left(t\right)}\;,$$

where *T* is the maximum time of the simulation, τ is the time period used as a parameter of the survival probability (a time window after which the probability of a molecule to remain in the cavity is determined), N(t)  is the number of molecules inside the α,δ-SCPN at the time t, and N(t, t+τ) is the remaining number of those molecules at t to τ. From this relation, the residence half-life time ($\tau_{1/2}$) can be estimated as the value of τ at P(τ) = 0.5.

The average velocity of waters and ions over time windows of 10 ps, along the whole trajectories (discarding the first 20 ns), was determined from the coordinates of the different species between frames stores in the trajectories. These velocities were associated to each initial position. Given the quasicylindrical geometry of the nanotube, the probability of the positions for water and ions was plotted using cylindrical coordinates (R,θ), where R is the distance from the axis of the nanotube and θ is the angle around such axis. The velocities of the same particles were also expressed in cylindrical coordinates and represented as a function of R or θ.

## Results and Discussion

### Stability of the α,δ-Self-Assembling Cyclic Peptide Nanotubes: The Fortress of the Nanotunnel

**FIGURE 2:**
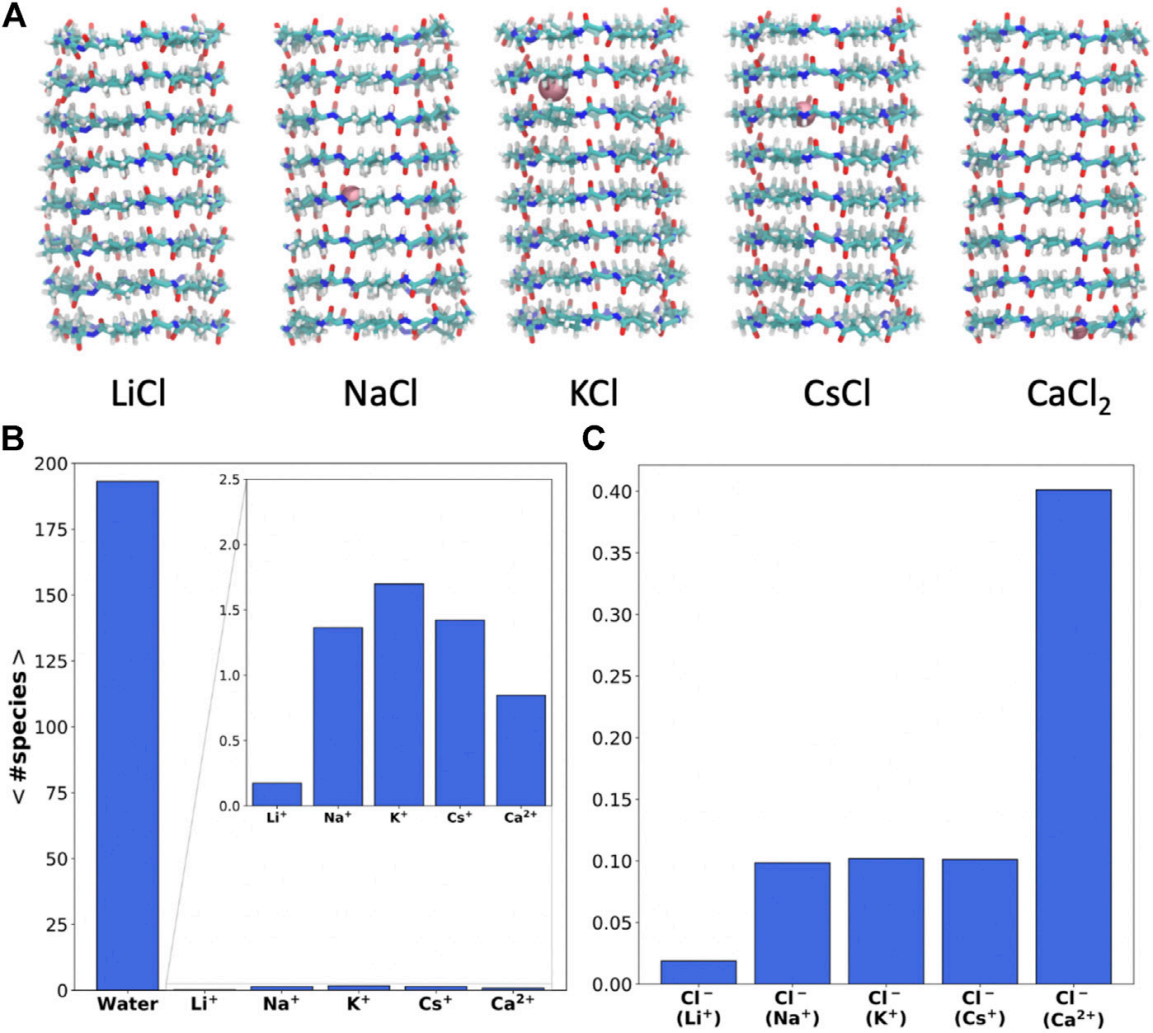
**(A)** Snapshot of the SCPNs corresponding to the last frame of the MD simulations (t = 200 ns), including the ions inside (pink) at that time (the lateral chain is not shown for clarity of the image). **(B and C)** Average number of cations **(B)** and anions **(C)** inside the α,δ-SCPN averaged over the last 180 ns of the MD simulations in LiCl, NaCl, KCl, CsCl, and CaCl_2_ 0.15 M, respectively. Diving into confinement: structure of water and ions inside the α,δ-SCPN.

Previous studies with an α,δ-SCPN composed by c-[*L*-Gln-δ-Ach-*D*-Trp-δ-Ach-*L*-Trp-δ-Ach-*D*-Leu-δ-Ach-*L*-Trp-δ-Ach-*D*-Trp-δ-Ach] showed that the nanotube structure is stable in the lipid bilayer environment (Calvelo et al., 2021). The substitution of both Gln and Leu by Trp residues shown in the present work does not have a significant effect on the transmembrane channel stability, as indicated by the low average Root Mean Square Deviations (RMSDs) of the SCPN backbone reached along the trajectories (Supplementary Table 1). The tubular shape is preserved in all the simulations (Figure 2A). The high and constant number of H-bonds between CPs along the channel backbone (Supplementary Table 1) confer rigidity to the whole structure, thus providing a stable scaffold throughout the α,δ-SCPN. The average number of H-bonds between CPs (78 in all the simulations) is slightly lower than the maximum that can be theoretically formed (12 H-bonds by each CP-pair, giving a total of 84 H-bonds). This is possibly due to some transient imperfections in the structure and also to the competition with the water molecules inside and outside the channel (Supplementary Table 1), which suggests the dynamic character of the tubular structure.

Despite its significant hydrophobic character, a considerable amount of water (∼195 molecules) can be found inside the α,δ-SCPN (Figure 2B). The amount of water within the nanocylinder volume delimited by the CPs is not significantly altered in the presence of ions (Supplementary Figure 1). On the other hand, whereas almost no Li^+^ cations can penetrate the pore, the α,δ-SCPN admits an average of 1–3 Na^+^, K^+^, or Cs^+^ and 1–2 Ca^2+^ cations (Figure 2B and Supplementary Figure 2A). The appearance of Cl^−^ anions inside the channels is significant but much lower than that of cations, and no more than a single anion was observed in this volume at the same time (Supplementary Figure 2B). In contrast, no anions at all were observed within the pore of the smaller and less hydrophobic α,γ-SCPNs (García-Fandiño et al., 2012a; Calvelo et al., 2015).

**FIGURE 3:**
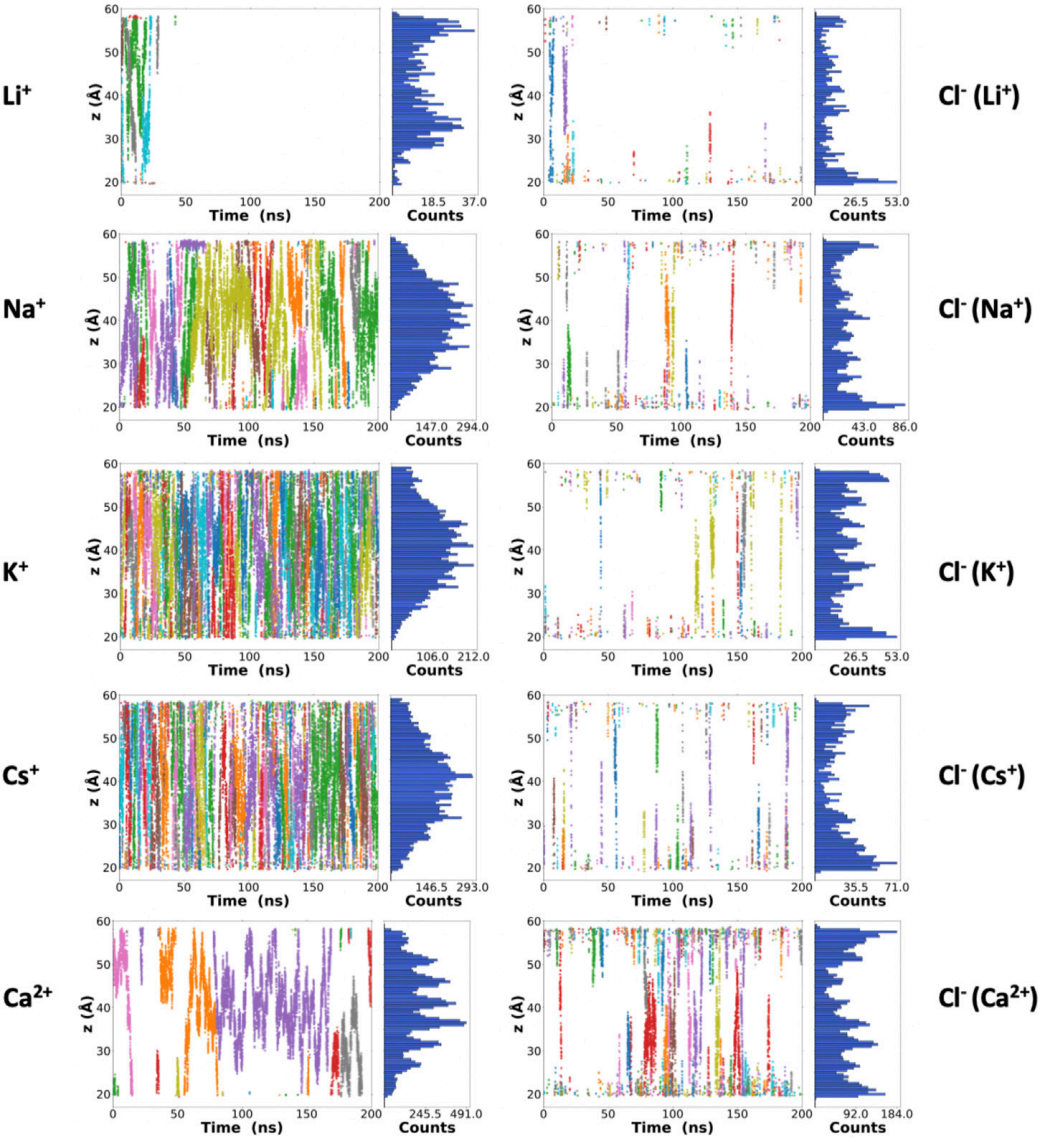
Z-coordinates (along the symmetry axis of the nanotube) for the cations **(left)** and anions **(right)** along the 200 ns of the corresponding trajectories. Each color represents a different atom as a function of time. From top to bottom: LiCl, NaCl, KCl, CsCl, and CaCl_2_ 0.15 M. A complementary plot, with the number of frames where at least one ion is located in each z-position of the nanotube, is also shown at the right side of each graphic.

Besides the number of ions inside the channels, it is possible to monitor their positions along the lumen of the corresponding nanotubes for each frame of the trajectory (Figure 3). Some Li^+^ cations were observed inside the α,δ-SCPN just for the first 25 ns of simulation. After this time, they appear attached to the lipid phosphates, which prevent their entry into the pore (Supplementary Figure 3). The astounding preference for phosphate groups displayed by Li^+^ ions is in good concordance with previous studies (Kruczek et al., 2017) that predict this behavior. This cation is sequestered by the polar head groups of the lipids in the membrane, thus rendering it unable to traverse the nanotube, at least at the time scale these experiments were carried out. Thus, Li^+^ cations in the nanopore cavity volume will not be considered for the rest of the analysis in this manuscript. As it had been previously observed for this type of channel (Calvelo et al., 2021) and analogously to α,γ-SCPNs (García-Fandiño et al., 2012a; Garcia-Fandiño et al., 2016), the effective radius is situated in the plane of the CP, whereas the maximum radius is located in the region between the two planes of the rings. However, the transport of Na^+^, K^+^, and Cs^+^ is significantly different to that observed in octameric α,γ-SCPNs (García-Fandiño et al., 2012a; Calvelo et al., 2015), presumably due not only to the higher hydrophobic character of this nanotube but also to larger radius of these three ions. While the pores formed by α,γ-SCPNs exhibit regions where the cations spend more time, acting as brakes against the advance of the ions along the pore, the transport of Na^+^, K^+^, and Cs^+^ in α,δ-SCPN takes place in a more continuous way, with a probability distribution centered in the middle of the nanotube (Figure 3). The discrete advance of the cation is still present in the case of Ca^2+^, where its accumulation in specific zones along the symmetry axis of the channel is much more noticeable than for the rest of the cations. Differences in the distribution functions along the main axis of the nanotube arise from the alternation between the smallest radii, corresponding to the plane of the CPs, and the larger radii regions, corresponding to the interplane sections.

The highest abundance of Cl^−^ inside the channel is observed for the case of the simulation with CaCl_2_. For this simulation, a single cation remains inside the α,δ-SCPN for long periods of more than 100 ns (Figure 2C and Figure 3). The distributions shown in Figure 3 indicate that a complementary Cl^−^ anion seems to accompany always the Ca^2+^ cations, suggesting that electrostatic interactions mediate the permeability of the nanotube toward anions. For the rest of the simulations, Cl^−^ gets only ever so shyly inside the nanotube, also sketching a relatively spatial complementarity along the symmetry axis of the nanotube with respect to the different countercations.

**FIGURE 4:**
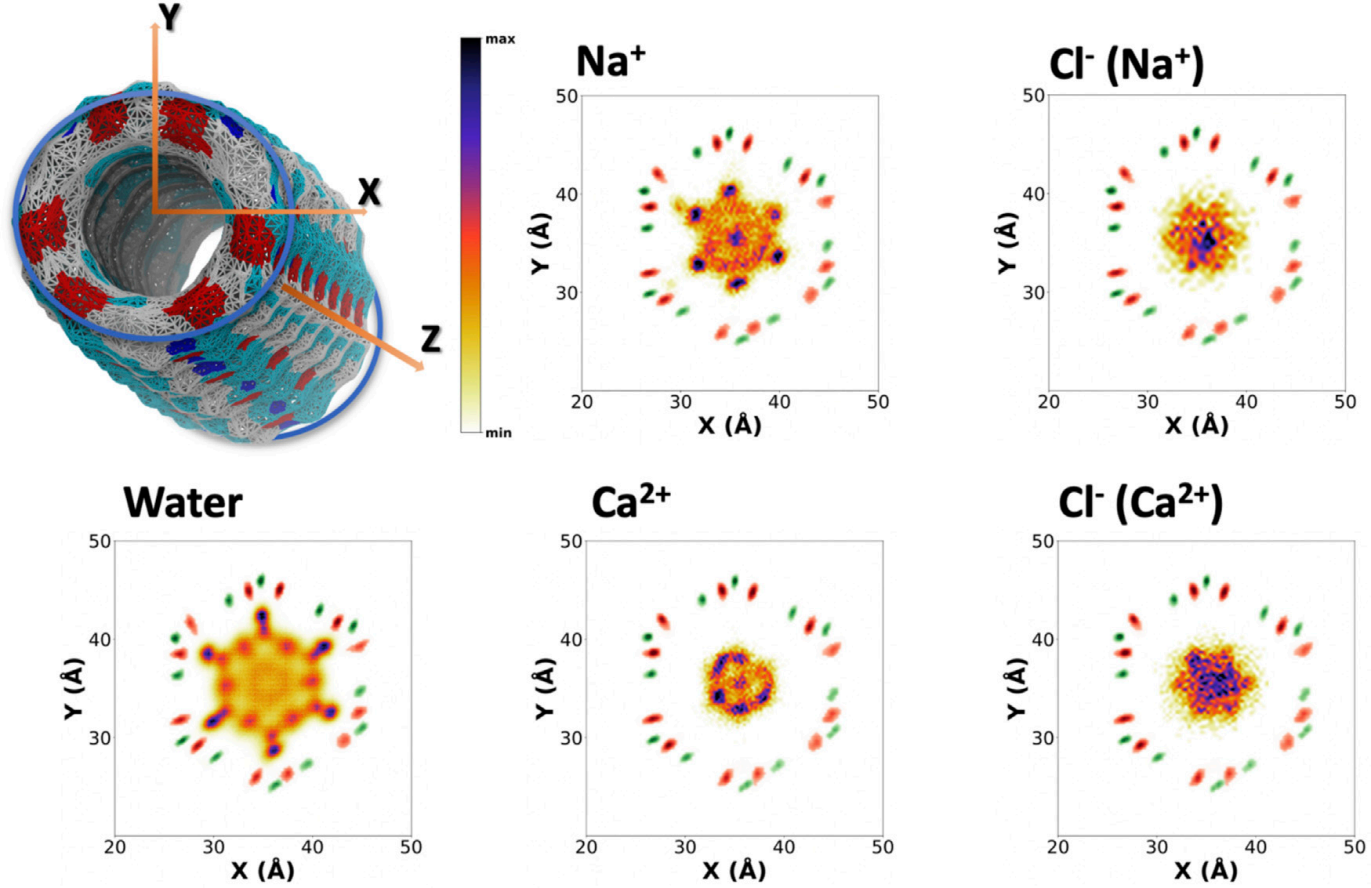
Positional probability distributions represented as heatmaps (increasing from orange to blue) at the XY plane for Na^+^, Ca^2+^, and their correspondent counter Cl^−^ anions. Water (from the blank trajectory) is shown at the down left of the figure. The averaged position of alpha carbons (green) and carbonyl groups (red) is also represented as a reference. These 2D-heatmaps were generated using the last 180 ns of the MD simulations.

**FIGURE 5:**
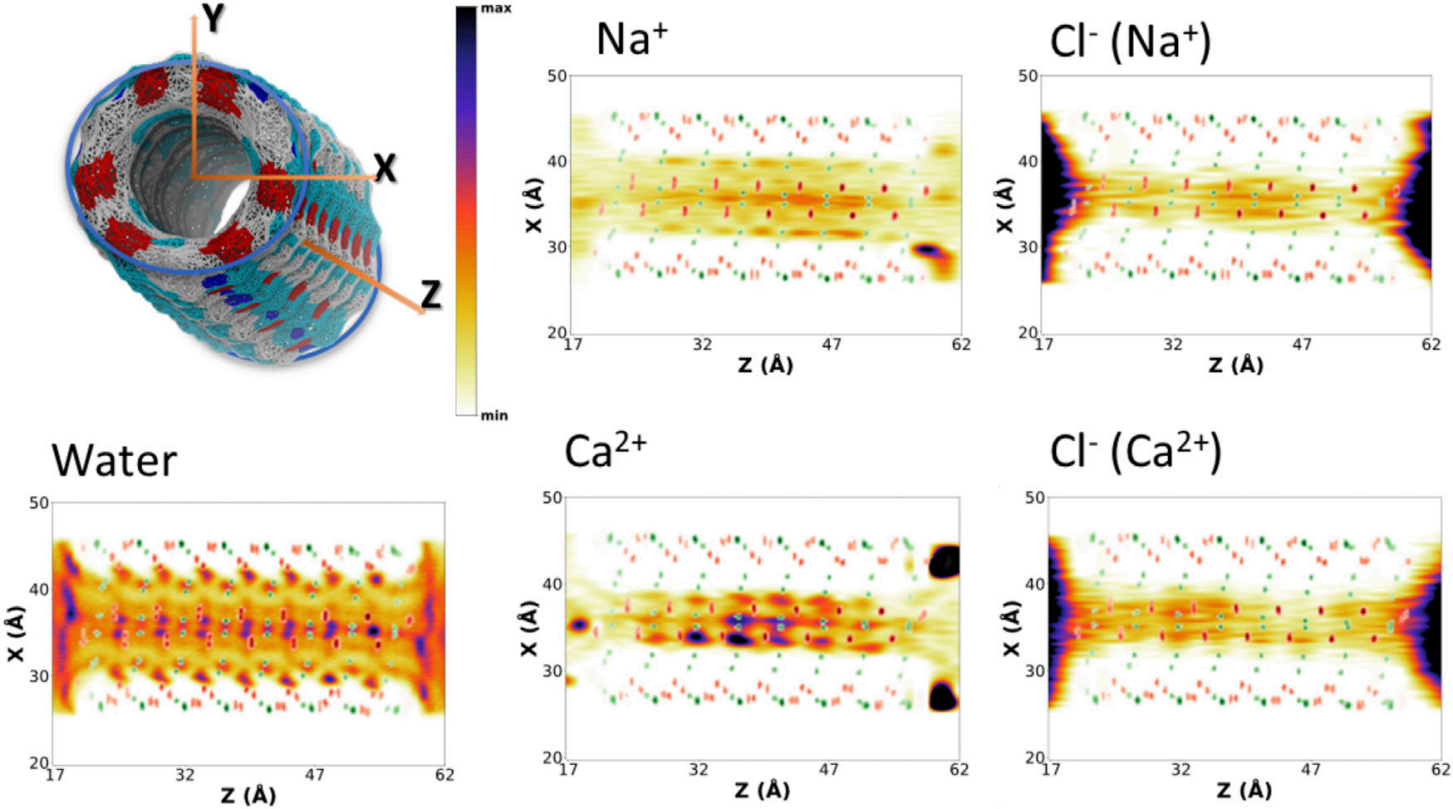
Positional probability distributions represented as heatmaps (increasing from orange to blue) at the XZ plane for Na^+^, Ca^2+^, and their correspondent counter Cl^−^ anions. Water (from the blank trajectory) is shown at the down left of the figure. The averaged position of alpha carbons (green) and carbonyl groups (red) is also represented as a reference. These 2D-heatmaps were generated using the last 180 ns of the MD simulations.

To better understand the distribution of species along the α,δ-SCPN, their probability distribution in two dimensions, obtained from the number of counts at each position with respect to the total amount of counts inside the SCPN, is represented as heatmaps. These 2D heatmaps have been represented in the transversal XY (Figure 4and Supplementary Figure 4) and longitudinal XZ (Figure 5 and Supplementary Figure 5) or YZ (Supplementary Figure 6) planes. Animations where the view of the heatmap is rotated along the longitudinal plane are available in the supplementary information (Supplementary Animation 1). Despite their larger diameter, compared to α,γ-SCPNs (García-Fandiño et al., 2012a; Calvelo et al., 2015), the nanoconfined water molecules in the α,δ-SCPN adopt a highly ordered distribution both in the transversal and longitudinal planes. The water spatial distribution over the transversal XY plane reveals a clearly defined six-point star shape, for the blank simulation (Figure 4). It is comprised of concentrical rings where water molecules tend to accumulate. The external ring, forming the tips of the star, corresponds to water directly attached to the CO and NH groups of the SCPN backbone *via* H-bonds (*type I,* from now on). In particular, most of these water molecules establish H-bonds with the CO groups of Trp (42 ± 3 H-bonds) and δ-Ach (18 ± 3 H-bonds) residues and to a lesser extent with the NH moiety (5 ± 1 H-bonds with the NH group of Trp residues and 6 ± 2 H-bonds with the NH group of δ-Achresidues) (Values Averaged from the Last 50 Ns of the MD Simulations). The following inner ring can be split into two types of water molecules: those bridged to the outer ring (*type II*) and those lying between the former water molecules, in the region next to the two methylene groups of the δ-Ach (*type III*). Finally, there is an innermost ring, more diffuse than those previously described (*type IV*). Furthermore, it is worth noticing that, besides the appearance of lobes where water tends to reside, there are regions that water tends to avoid, creating channels with decreased water density. These regions guide the position and diffusion of cations (see below). Water distribution on the XZ and YZ planes is also very well defined (Figure 5 and Supplementary Figure 6). The darkest lobes correspond to inter CP plane regions, coexisting with slightly less denser areas belonging to water placed at the CP planes.

The presence of ions in the simulation does not seem to affect the exotic water pattern found in the blank simulation (Supplementary Figure 7). Instead, it seems that this predefined water distribution inside the α,δ-SCPN creates a coordinating scaffold through which the ions move. In this way, the 2D heatmaps for cations in the XY plane are complementary to those corresponding to water (Figure 4 and Supplementary Figure 4). Similar to water, ions also exhibit a characteristic 2D distribution heatmap over the SCPN circular plane. Na^+^ forms a reciprocal hexagonal star, with six peaks at the regions in contact with *type II* and *type III* waters described before (Figure 4). Overall, the shape of the star becomes more diffuse from Na^+^ to Cs^+^ (Figure 4 and Supplementary Figure 4), a demeanor possibly attributed to the larger ion radius. Thus, bigger cations prefer the central zones of the pore, whereas those smaller are more localized at the borders or inside specific channels where they can fit better. On the other hand, Ca^2+^ illustrates the influence of charge, rather than radius (as compared with the monovalent cations), on the preferred position of a divalent cation through an α,δ-SCPN (Figure 4). The probability distribution of the divalent cation displays a compact shape in the XY plane, indicating that the ions are confined in the innermost part of the lumen. This suggests that Ca^2+^ ion movements are restricted to smaller regions than the other cations. The ion disposition along the symmetry axis of the nanopore is also different, depending on its size and charge. While the most populated regions for Na^+^ and K^+^ are those between CP planes, Cs^+^ and Ca^2+^ seem to prefer the plane of the CP (Figure 5 and Supplementary Figures 5,6).

The 2D heatmaps for Cl^−^ suggest a dependence of the anion location and diffusion on their cation counterpart. Cl^−^ accumulation in the XY plane reveals a less-defined six-point star, the clearest case being CaCl_2_ (Figure 4). It is notable how these stars are rotated 60^°^ with respect to their cation analogues. While positive charges have well-defined channels through which they can diffuse without disrupting the scaffold that water creates inside the SCPN and may even get “lubricated” by coordinating to their oxygens, anions have trouble finding a place to fit and move through, even more so if they are as bulky as a chloride. On top of that steric hindrance, the only possible coordination that a Cl^−^ can have with water is through its hydrogens. This further disrupts the water scaffold, as it would imply an inversion of the conformation of some water molecules. This flip would render the oxygen atom from the coordinated water molecule close to the oxygen atoms of the waters forming the scaffold, creating an electrostatic repulsion, and destabilizing a very stable lattice by entailing an energetic penalty. On the other hand, the anions’ 2D heatmaps of the averaged positions along the longitudinal planes show very similar profiles for all the simulations, the only difference being the intensity of the population (Figure 5 and Supplementary Figures 5,6).

**FIGURE 6:**
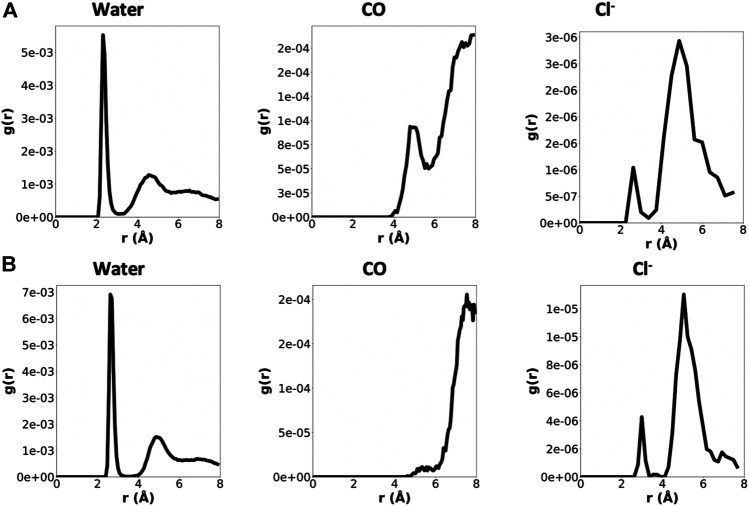
RDFs for Na^+^ **(A)** and Ca^2+^ **(B)** considering different contributions (water, CO groups, and Cl^−^ ions), normalized by the number of cations and number of contributions. Just the last 180 ns were considered for the analysis. Note that the scales for the three contributions are not the same.

In order to gather information about the surroundings of each cation within the α,δ-SCPN, Radial Distribution Functions (RDFs) were calculated (Figure 6 and Supplementary Figure 8). They display a clear first water coordination sphere for all species, which migrates toward bigger distances for ions presenting larger atomic radii. None of the cations studied tend to directly coordinate to the CO groups of the amino acid backbone in this first coordination sphere at distances ∼4–6 Å. Except for Ca^2+^, these CO groups do participate in their second coordination sphere. On the other hand, some Cl^−^ ions do directly contribute to the first coordination sphere of all the cations, but this contribution is poor.

**FIGURE 7:**
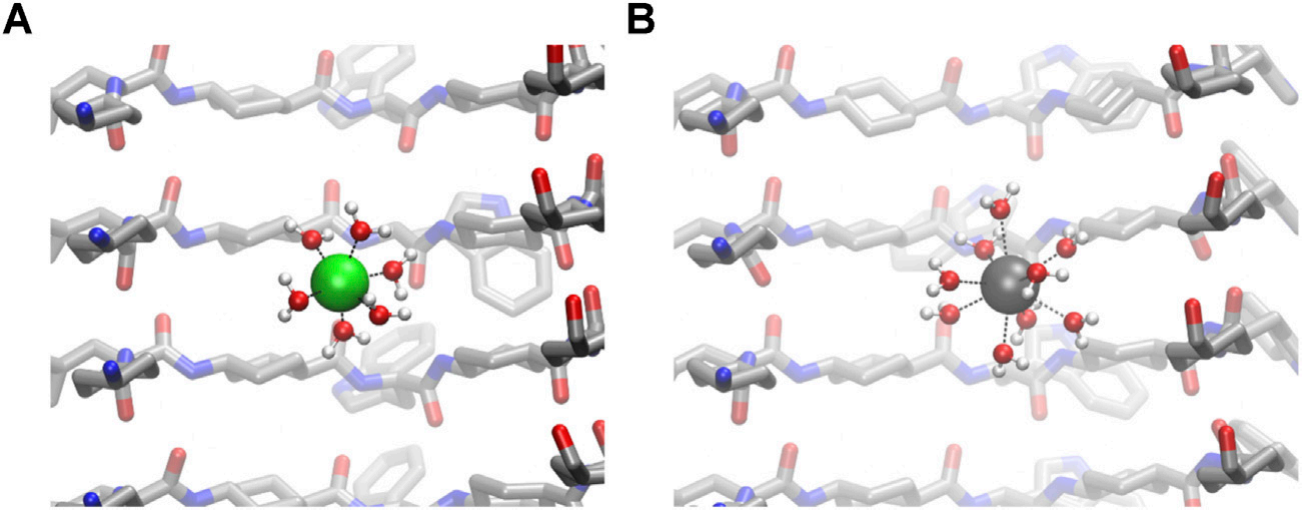
Details of the first sphere of coordination of confined Na^+^ **(A)** and Ca^2+^ **(B)**.

The calculation of the number of contacts between cations and their possible contributions to the first coordination sphere (calculated from the RDF values) also confirms that CO groups do not coordinate directly to cations (Figure 7). The number of water molecules in the first coordination sphere of all cations is very similar to the theoretical maximum in bulk, suggesting that the energetic penalty that these ions pay to enter into the tube is low (Table 1 and Figure 7) (Ohtomo and Arakawa, 1979; Bakó et al., 2002; Varma and Rempe, 2006). Almost no Cl^−^ ion or oxygen from CO was found at the first coordination sphere distance of any cation, confirming that this situation is unlikely.

**TABLE 1:** Average number (and standard deviation) of contacts between water, oxygens from CO, and Cl^−^ anions participating in the first coordination sphere of each cation. The radius of the first coordination sphere was taken from the RDF calculations. Just the last 180 ns were considered for the analysis.

Cation	Water	CO	Cl^−^
Na^+^	5.5 ± 0.56	0.00 ± 0.03	0.00 ± 0.04
K^+^	6.21 ± 0.75	0.01 ± 0.13	0.01 ± 0.08
Cs^+^	5.26 ± 0.94	0.00 ± 0.08	0.01 ± 0.11
Ca^2+^	8.83 ± 0.4	0.00 ± 0.01	0.01 ± 0.10

It was observed that the number of coordinating water molecules inside the nanotube often exceeds the theoretical maximum (6) for the cations. This situation is clear for K^+^, where the average number is 6.21 ± 0.75. One plausible explanation is that nanoconfined systems behave differently than their bulk relatives. SCPNs can be approximated to a hollow cylinder, where one dimension (vertical, Z) is much larger than the other two (circular plane, XY). This leaves a space where two degrees of freedom become restricted, and thus, movement in those directions becomes hampered. It is easy to imagine, then, that in such a system with particles moving inside, collisions are more likely to occur than in a free unrestricted medium. This might lead to a compression of the first sphere of coordination for cations, forcing the ligands toward the metallic center and affecting the ligand exchange process. It should also be noted by the higher SDs on the inner number of waters (Table 1), when compared with those of the bulk analysis, that these “hypercoordinated” states might only happen for very short periods of time, being unstable.

### Movement Into Confinement: Dynamics of Water and Ions Inside the α,δ-Self-Assembling Cyclic Peptide Nanotubes

For the proper characterization of the α,δ-SCPN as a transmembrane channel, it is interesting to study how the different molecules move through the nanotube, in addition to characterize their most likely location. From the survival probability (Supplementary Figure 9) and the corresponding half-life time values (Table 2), it can be appreciated that the most short-lived species in the α,δ-SCPN is water, followed by Cs^+^, K^+^, Na^+^, and Ca^2+^. The residence half-time for Cl^−^ anions is significantly lower, even for the simulation in CaCl_2_.

**TABLE 2:** Residence half-life time ($\tau_{1/2}$) (ns) calculated from the survival probability [P(τ)] evolution for cations and anions in each simulation (Supplementary Figure 9).

$\tau_{1/2}\;\left(ns\right)$	Cation	Anion
NaCl	3.1	0.2
KCl	1.2	0.2
CsCl	1.0	0.2
CaCl_2_	4.6	0.3

**FIGURE 8:**
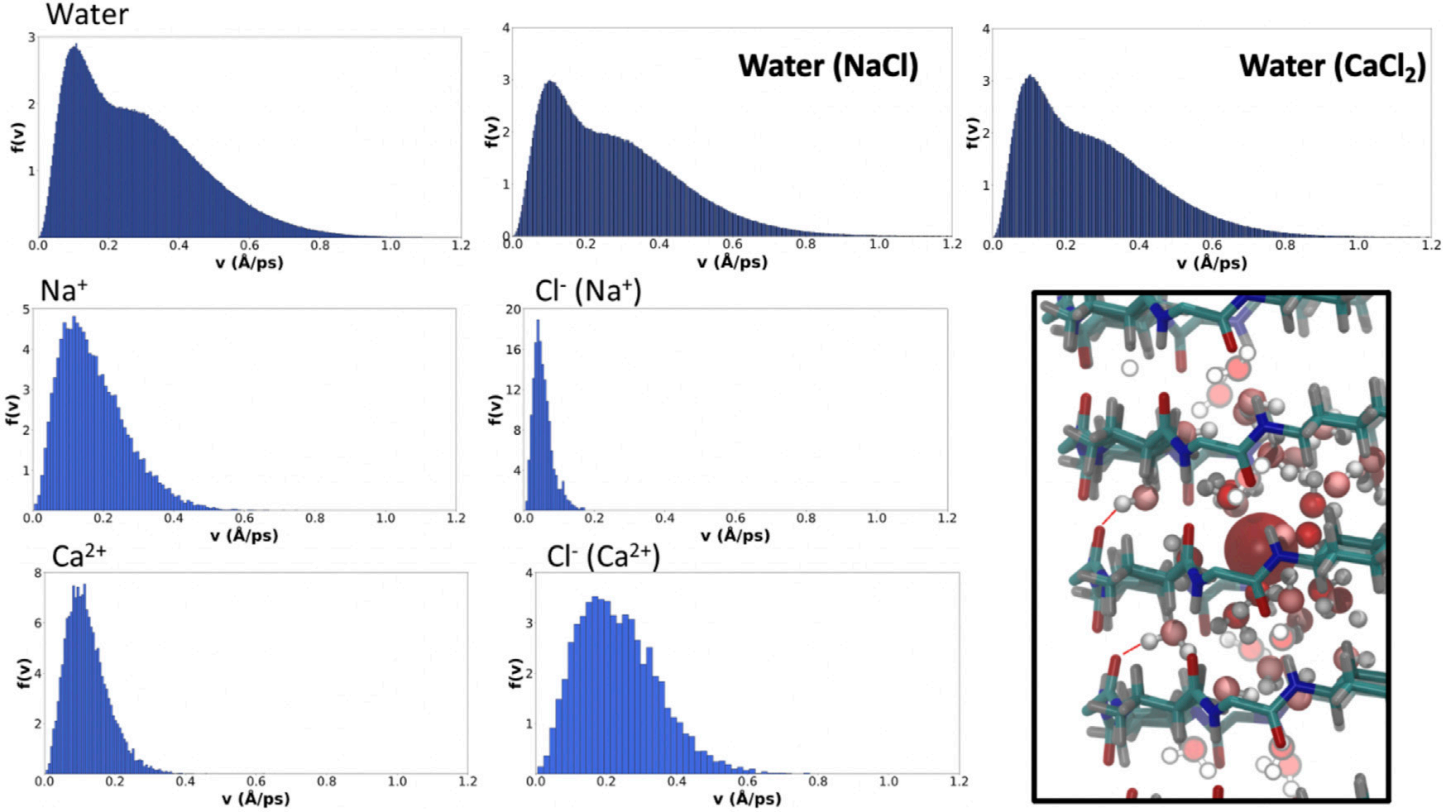
Velocity probability distributions for water (top row, for blank and simulations in the presence of NaCl and CaCl_2_) and the selected ions. Bin-width has been selected applying the Freedman–Diaconis rule (Freedman and Diaconis, 1981). In the right-bottom square box, a representation of the coordination of Ca^2+^ with waters from the first and second coordination spheres is shown.

The distributions of average velocities between saved frames (10 ps far from each other) of the different ions and water molecules were determined from the MD trajectories. The results for the solvent and ions show that water molecules reach the highest average speeds over this windows time, although there is a significant contribution indicating the existence of slower water molecules in the corresponding distribution, presumably those involved in *type I* H-bonds (Figure 8). Just as the presence of ions did not seem to affect the overall water pattern inside the pore, they do not seem to alter their velocity distribution either. For a given valence, cations with bigger radii tend to diffuse faster along the α,δ-SCPN, as it can be observed from the broadening toward higher speeds of the distributions from Na^+^ to Cs^+^ (Figure 8 and Supplementary Figure 10). Cation speed seems to be directly related to its degree of accommodation in the nanotube cavity. Na^+^, for example, seems to be relatively comfortable inside the pore, compared to the other ions, as can be inferred from the ordered 2D heatmap pattern corresponding to the XY plane (Figure 4). The spatial disposition into a full set of lobes in each of the points of the star and an extra central lobe allows this ion to find several places where its complete coordination sphere can be maintained without disturbing the general order of confined water. The alternation among these positions in the XY plane seems to slow down its movement along the nanotube symmetry axis, justifying its longer residence times. On the other hand, whereas K^+^ and Cs^+^ display roughly the same amount of coordinating waters than Na^+^ (Table 1), their bigger radii demand a global larger space inside the pore. This hinders the ability to reach a stable scaffold of H-bonds with the confined water in the channel and forces it to move around less favorable locations, thus promoting the diffusion through the nanotube. This is also in agreement with the more diffuse star-like shapes these two ions present in the XY planes compared to Na^+^ (Figure 4). The comparison of the results for Ca^2+^ and Na^+^ (Figure 8) provides evidence of the important role of the charge over the radius or mass of the cation. Both ions have a very similar radii, but the charge of the Ca^2+^ forces its coordination sphere to accommodate a larger number of water molecules (Figure 7), thus extending it to longer distances. Despite being restricted to a more central position in the nanotube, thus suggesting a lower interaction with the nanotube walls, Ca^2+^ exhibits the higher residence times of all the studied cations (Table 2 and Supplementary Figure 9). This apparent contradiction is, nonetheless, supported by the fact that Ca^2+^, together with its set of coordinate water molecules, fits better at the plane of the CPs than between CP planes. In that arrangement, the second coordination sphere is composed just by waters, as confirmed by the RDF and coordination numbers. Many of these water molecules are of *type I*, thus coordinated to CO groups from the SCPN backbone (Figure 8, square).

The velocity distribution for Cl^−^ is similar for all the monovalent cation simulations, much lower than those corresponding to the cationic counterparts (Figure 8). This fact, together with their short residence times in the nanotube, explains the fact that they hardly explore any position in the XY plane in most of the cases (Figure 4 and Supplementary Figure 4), crossing the channel in a very short time. It is again in the simulation with Ca^2+^ where a remarkably different demeanor is observed in the movement of the Cl^−^ anion (Figure 8). In this case, not only the distribution is broader and with a slower decay rate than in the rest of the simulations but it also reaches higher speeds than the cation compared to the rest of cases. Their higher speed is also accompanied by a slightly longer residence time (Table 2 and Supplementary Figure 9). The explanation for this difference relies on the influence that the counterion has in the movement of Cl^−^. Although it does not occupy the Ca^2+^ first coordination sphere, its movement is coupled to the correspondent cation, having an influence in the diffusion along the nanotube.

**FIGURE 9:**
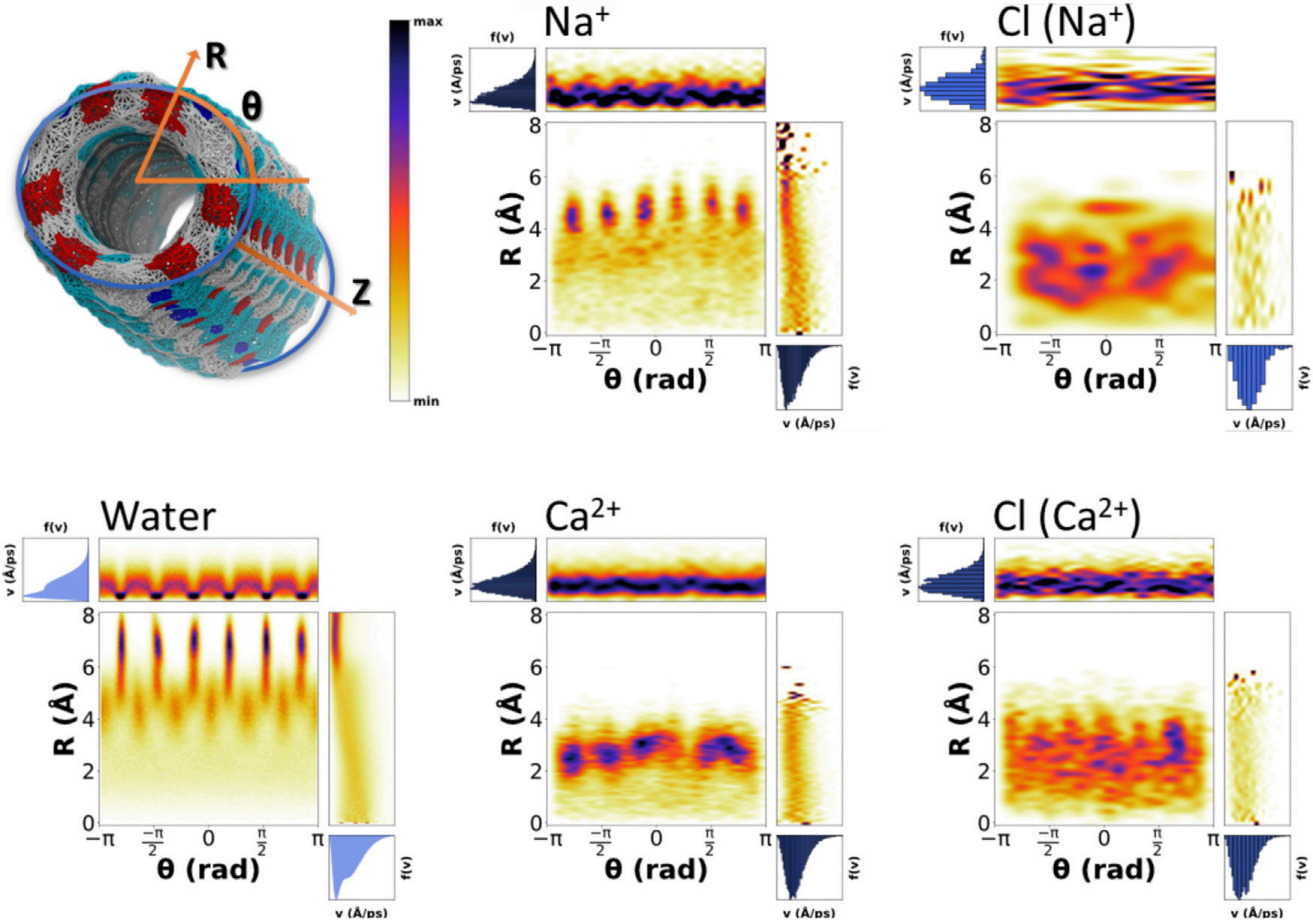
Position–velocity correlation diagrams for water (bottom left), selected cations (middle top, Na^+^, and down, Ca^2+^), and correspondent counterions (right column). The central plot of each diagram corresponds to the probability of positions as a function of cylindrical coordinates (R,θ), the top plot is the velocity distribution as a function of the angle, and the right plot is the velocity distribution as a function of the distance to the center of the nanotube symmetry axis. The analysis was generated using the last 180 ns of the MD simulations.

The existence of more than one possible velocity distributions for one single species suggests that there should be more than one associated behaviors. We propose that those different conducts should be related to the position of the particle inside the nanotube. The velocity of particles located in the inner part of the nanotube is expected to be different from that located in one of the lobes. To demonstrate this hypothesis and taking advantage of the tubular shape of the nanopore, 2D heatmap distributions of positions and velocities in cylindrical coordinates (R,θ) were created (Figure 9 and Supplementary Figure 11). The velocity distributions for both the water molecules and cations display a clear correlation with the distance to center of the pore (R). There is also some dependence on θ, although to a lesser extent, generally corresponding to the slower motion at the lobes near the walls of the SCPN. Water, which displays a much better sampling than ions, gives the most detailed picture. It is possible to appreciate that the outermost lobes, from 6–8 Å, correspond to the slowest molecules (*type I*). Out of this area, the region from 5–6 Å exhibits slightly higher velocities with a broader span. This corresponds to the *type II* waters of the middle ring, defined in the previous section. In the region from 3–6 Å and intercalated between the lobes in the θ-axis, the *type III* waters of the middle ring are observed. These water molecules reach higher velocities, and the shape of the distribution is narrower than that of the *type IV* waters. Finally, corresponding to the innermost region of the SCPN, a change in slope in the R correlation diagram, from 3 Å and down, is seen. This indicates that the water molecules falling inside this region will move faster than the previous ones, and the dependence on R is more pronounced than for the previously described waters.

Again, Na^+^ (Figure 9), K^+^, and Cs^+^ (Supplementary Figure 10) seem to present similarities in their behavior. For K^+^ and Cs^+^, the dependence of the velocities on R is clear, displaying a confined slow regime in the lobes and a tendency for velocity to increase as we get closer to the center of the SCPN. Na^+^ displays the same behavior, although the dependence with R is weaker. Also, its innermost region seems to have a drop on the velocities, possibly due to its central high-density region (Figure 4). In line with other analysis, Ca^2+^ (Figure 9) also displays a completely different behavior. The correlation between the speeds and R is not as clear as for the other cations, but a sort of discontinuity in the velocity as a function of R can be observed around 1.75 Å (Figure 9). Also, the relationship between θ and the velocity distribution seems to be slightly smoother than for the rest of the cations.

The velocity distributions of anions are similar for all the simulations. Cl^−^ movement does not clearly depend on its position inside the nanotube (Figure 9 and Supplementary Figure 11). Only the case for the simulation with CaCl_2_ seems to have a small drift toward slightly higher speeds for the smallest values of R. This behavior is in agreement with what was previously proposed about the movement of the anions: their diffusion along the nanotube is not free, but it is driven by the cations present in the system. Thus, the transport of Cl^−^ by cations with the same charge will be very similar to each other, while changing the valence of the cation a high impact on the velocities reached by the anion is expected, compared to that caused by the positional distribution within the SCPN.

## Conclusion

The observations of an underwater expedition through the nanoconfined space of a transmembrane α,δ-SCPN by wearing a diving equipment in the form of MD simulations are described here. The presence of δ-aminocycloalkanecarboxylic acids in the CP sequence confers a partially hydrophobic character to the pore, which has a critical impact on the behavior of the nanoconfined ions and water molecules. MD simulations of an α,δ-SCPN composed of CPs with the sequence *c*-[*L*-(Trp-δ-Ach)-(*D*-Trp-δ-Ach)-*L*-(Trp-δ-Ach)-*D*-(Trp-δ-Ach)-*L*-(Trp-δ-Ach)-*D*-(Trp-δ-Ach)] in the presence of different salt compositions (blank, LiCl, NaCl, KCl, CsCl, and CaCl_2_) indicate that they are stable when inserted into a lipid bilayer. The hybrid hydrophilic–hydrophobic environment created by the nature of the residues in the sequence defines the disposition of water molecules inside the channel. Nanoconfined water in the α,δ-SCPN adopts a highly ordered distribution both in the transversal and longitudinal planes. The water spatial distribution over the transversal plane reveals a clearly defined six-point star shape, formed by at least four water types (*type I–IV*), with a different diffusion behavior. While Li^+^ is sequestered by the lipid phosphate groups which prevent its internalization in the nanopore, Na^+^, K^+^, Cs^+^, and Ca^2+^ were observed to go inside the channel at different rates, proportions, and locations. Globally, no more than three ions of the same class appear within the channel at the same time in any of the simulations, the average number being between one and three in most cases. The appearance of Cl^−^ anions inside the channels is scarce compared to that of cations although, unlike happened with the smaller and less-hydrophobic α,γ-SCPNs, a small proportion was observed.

Ions accommodate inside the cylinder trying to disturb as little as possible the exotic pattern that water molecules create inside the channel. The ions with less impact in the water organization remain for a longer time inside the nanotube, as revealed by the survival probability profile and the corresponding residence half-life times. This is achieved in the first place by Ca^2+,^ and then by Na^+^, K^+^, and Cs^+^, in this order. In all cases, the first water coordination spheres for all the cations are completely preserved, being the type of molecules that belong to its second sphere of coordination that will define the advance of the cations along the symmetry axis of the nanotube.

## Data Availability

The datasets generated and analyzed for this study are included in the article/Supplementary Material. All initial coordinates, topologies and mdp files needed for reproducing these simulations are available in Zenodo through the following link: https://zenodo.org/record/4732313. Further inquiries can be directed to the corresponding author.
