# Supplementary material for: Transmembrane Self-Assembled Cyclic Peptide Nanotubes Based on α‐Residues and Cyclic δ‐Amino Acids: A Computational Study
Source: Front Chem. 2021 Jul 27;9:704160. doi: 10.3389/fchem.2021.704160 (PMC8353252; doi:10.3389/fchem.2021.704160)
Supplement: Supplementary file 1 [file DataSheet3.DOCX]

**Transmembrane self-assembled cyclic peptide nanotubes based on α‐residues and cyclic δ‐amino acids: a computational study**

**Alexandre Blanco-González^1,2^, Martín Calvelo^1^, Pablo F Garrido^2^, Manuel Amorín^1^, Juan R. Granja^1^, Ángel Piñeiro^2^, Rebeca Garcia-Fandino^1*^**

^1^Departamento de Química Orgánica, Center for Research in Biological Chemistry and Molecular Materials, Universidade de Santiago de Compostela, Campus Vida s/n, E-15782 Santiago de Compostela, Spain

^2^ Departamento de Física Aplicada, Facultade de Física, Universidade de Santiago de Compostela, E-15782 Santiago de Compostela, Spain

**Supporting Information**

Animations (III)


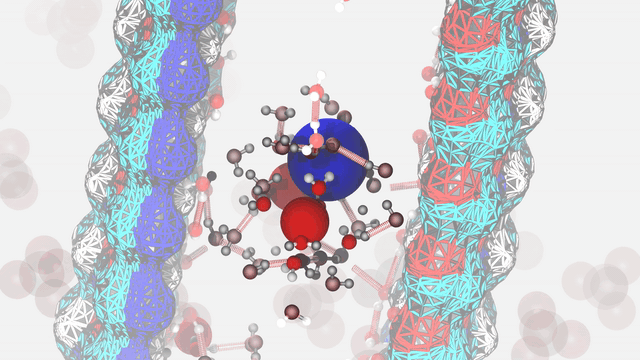


Animation 4: Depiction of two Na^+^ ions stabilizing a Cl^−^. The second coordination sphere of the cations mixes with the first coordination sphere of the anion, being this the possible mechanism for the introduction of negatively charged species inside the SCPN.


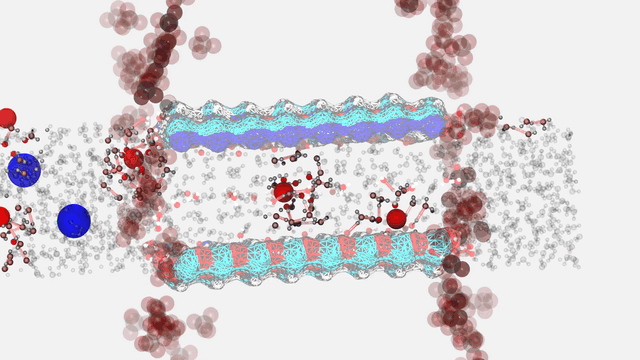


Animation 5. Extract from the CaCl_2_ simulation where two cations mediate the translocation of one anion through the SCPN, emphasizing the importance of positive charge for the transport of negatively charged molecules through the α,δ-SCPN.
