# Supplementary material for: Transmembrane Self-Assembled Cyclic Peptide Nanotubes Based on α‐Residues and Cyclic δ‐Amino Acids: A Computational Study
Source: Front Chem. 2021 Jul 27;9:704160. doi: 10.3389/fchem.2021.704160 (PMC8353252; doi:10.3389/fchem.2021.704160)
Supplement: Supplementary file 2 [file DataSheet2.DOCX]

**Transmembrane self-assembled cyclic peptide nanotubes based on α‐residues and cyclic δ‐amino acids: a computational study**

**Alexandre Blanco-González^1,2^, Martín Calvelo^1^, Pablo F Garrido^2^, Manuel Amorín^1^, Juan R. Granja^1^, Ángel Piñeiro^2^, Rebeca Garcia-Fandino^1*^**

^1^Departamento de Química Orgánica, Center for Research in Biological Chemistry and Molecular Materials, Universidade de Santiago de Compostela, Campus Vida s/n, E-15782 Santiago de Compostela, Spain

^2^ Departamento de Física Aplicada, Facultade de Física, Universidade de Santiago de Compostela, E-15782 Santiago de Compostela, Spain

**Animations (I)**


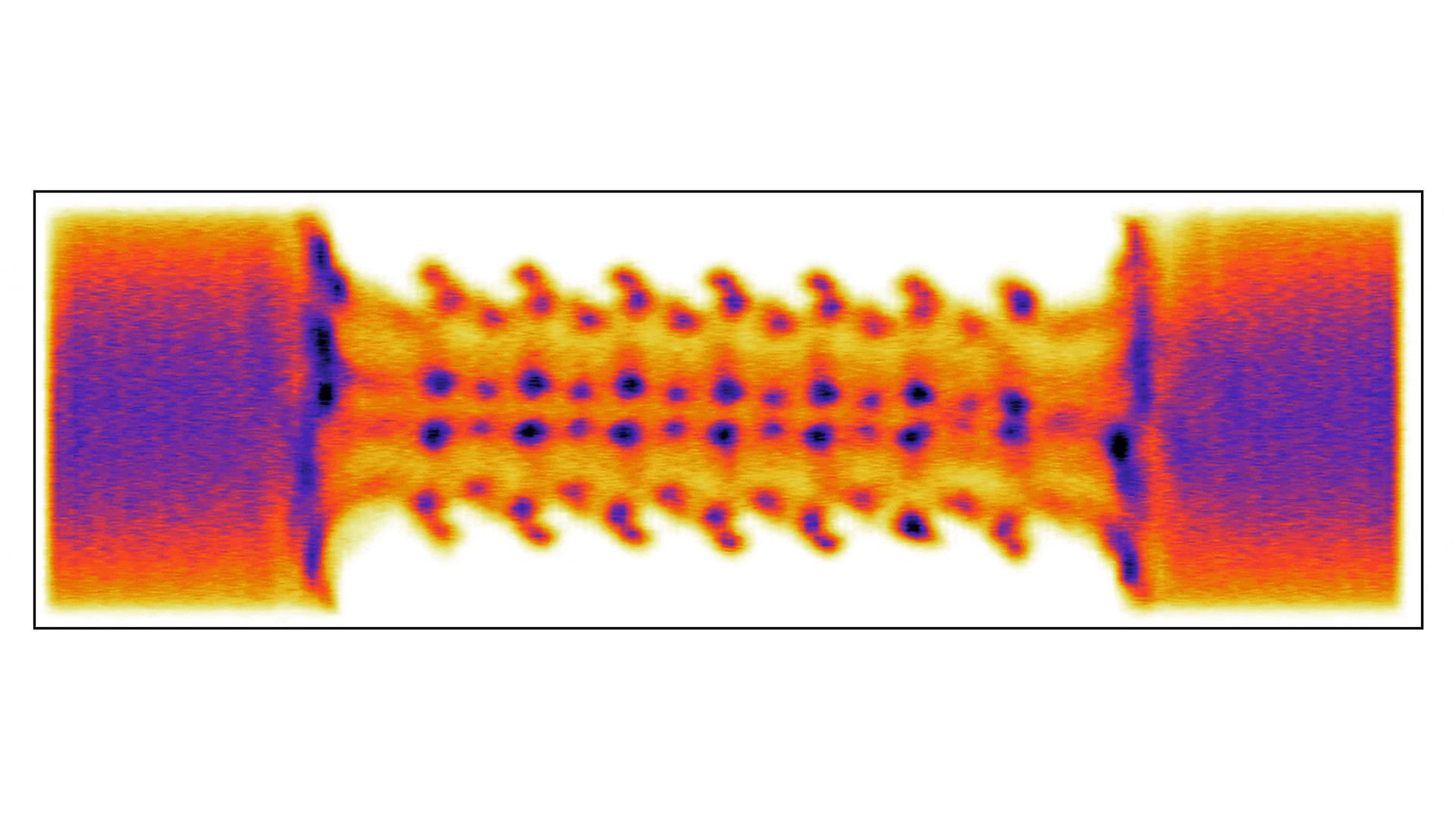


**Animation 1:** Rotating water heatmap along the main axis of the nanotube for the blank simulation (without ions).
